# Divergence of Legionella Effectors Reversing Conventional and Unconventional Ubiquitination

**DOI:** 10.3389/fcimb.2020.00448

**Published:** 2020-08-21

**Authors:** Tomoe Kitao, Hiroki Nagai, Tomoko Kubori

**Affiliations:** ^1^Department of Microbiology, Graduate School of Medicine, Gifu University, Gifu, Japan; ^2^G-CHAIN, Gifu University, Gifu, Japan

**Keywords:** *Legionella*, deubiquitinases, ubiquitin, effectors, metaeffectors, type IV secretion, *Legionella*-containing vacuoles, membrane traffic

## Abstract

The intracellular bacterial pathogen *Legionella pneumophila* employs bacteria-derived effector proteins in a variety of functions to exploit host cellular systems. The ubiquitination machinery constitutes a crucial eukaryotic system for the regulation of numerous cellular processes, and is a representative target for effector-mediated bacterial manipulation. *L*. *pneumophila* transports over 300 effector proteins into host cells through its Dot/Icm type IV secretion system. Among these, several effector proteins have been found to function as ubiquitin ligases, including unprecedented enzymes that catalyze ubiquitination through unconventional mechanisms. Recent studies have identified many *L*. *pneumophila* effector proteins that can interfere with ubiquitination. These effectors include proteins that are distantly related to the ovarian tumor protein superfamily described as deubiquitinases (DUBs), which regulate important signaling cascades in human cells. Intriguingly, *L*. *pneumophila* DUBs are not limited to enzymes that exhibit canonical DUB activity. Some *L*. *pneumophila* DUBs can catalyze the cleavage of the unconventional linkage between ubiquitin and substrates. Furthermore, novel mechanisms have been found that adversely affect the function of specific ubiquitin ligases; for instance, effector-mediated posttranslational modifications of ubiquitin ligases result in the inhibition of their activity. In the context of *L*. *pneumophila* infection, the existence of enzymes that reverse ubiquitination primarily relates to a fine tuning of biogenesis and remodeling of the *Legionella*-containing vacuole as a replicative niche. The complexity of the effector arrays reflects sophisticated strategies that bacteria have adopted to adapt their host environment and enable their survival in host cells. This review summarizes the current state of knowledge on the divergent mechanisms of the *L*. *pneumophila* effectors that can reverse ubiquitination, which is mediated by other effectors as well as the host ubiquitin machinery.

## Introduction

Ubiquitination is one of the most versatile posttranslational modifications found in eukaryotic cells, and it controls a wide array of cellular processes (Hershko and Ciechanover, 1998; Hochstrasser, 2009). *Legionella pneumophila*, the causative agent for Legionnaires' disease, which is a severe form of pneumonia, employs diverse strategies to utilize or modulate its host's ubiquitin system for its own benefit (Hubber et al., 2013; Lin and Machner, 2017; Qiu and Luo, 2017a,b; Kubori et al., 2019). This bacterium transports a vast array of effector proteins into the host cell cytosol via the type IV secretion system (T4SS), which is encoded by *dot/icm* genes in the bacterial genome (Nagai and Kubori, 2011; Kubori and Nagai, 2016; Galán and Waksman, 2018). Using the effector proteins, *L*. *pneumophila* establishes a replicative organelle called the *Legionella*-containing vacuole (LCV), counteracts host immune defense, and survives and replicates in host cells (Isberg et al., 2009; Hubber and Roy, 2010; Asrat et al., 2014). The LCV originates from the plasma membrane-derived phagosome, and is remodeled into an endoplasmic reticulum (ER)-associated compartment. In this way, the LCV co-opts several host factors to facilitate the recruitment and fusion of ER-derived vesicles by intercepting membrane traffic between the ER and the Golgi apparatus (Asrat et al., 2014; Hilbi et al., 2017). It has been shown that LCVs are highly decorated with ubiquitin through the Dot/Icm T4SS-dependent process, and a significant amount of ubiquitinated proteins are degraded by the host proteasome (Dorer et al., 2006). One possible explanation for ubiquitin accumulation on the bacterial vacuoles is that degraded ubiquitinated proteins serve as a source of nutrition for bacterial proliferation (Price et al., 2011). On the other hand, it has been well established that ubiquitinated proteins in cytosolic bacteria or bacterial vacuoles are readily recognized by host immune systems, including pathogen-associated selective autophagy, the commonly named xenophagy (Perrin et al., 2004).

**Figure 1:**
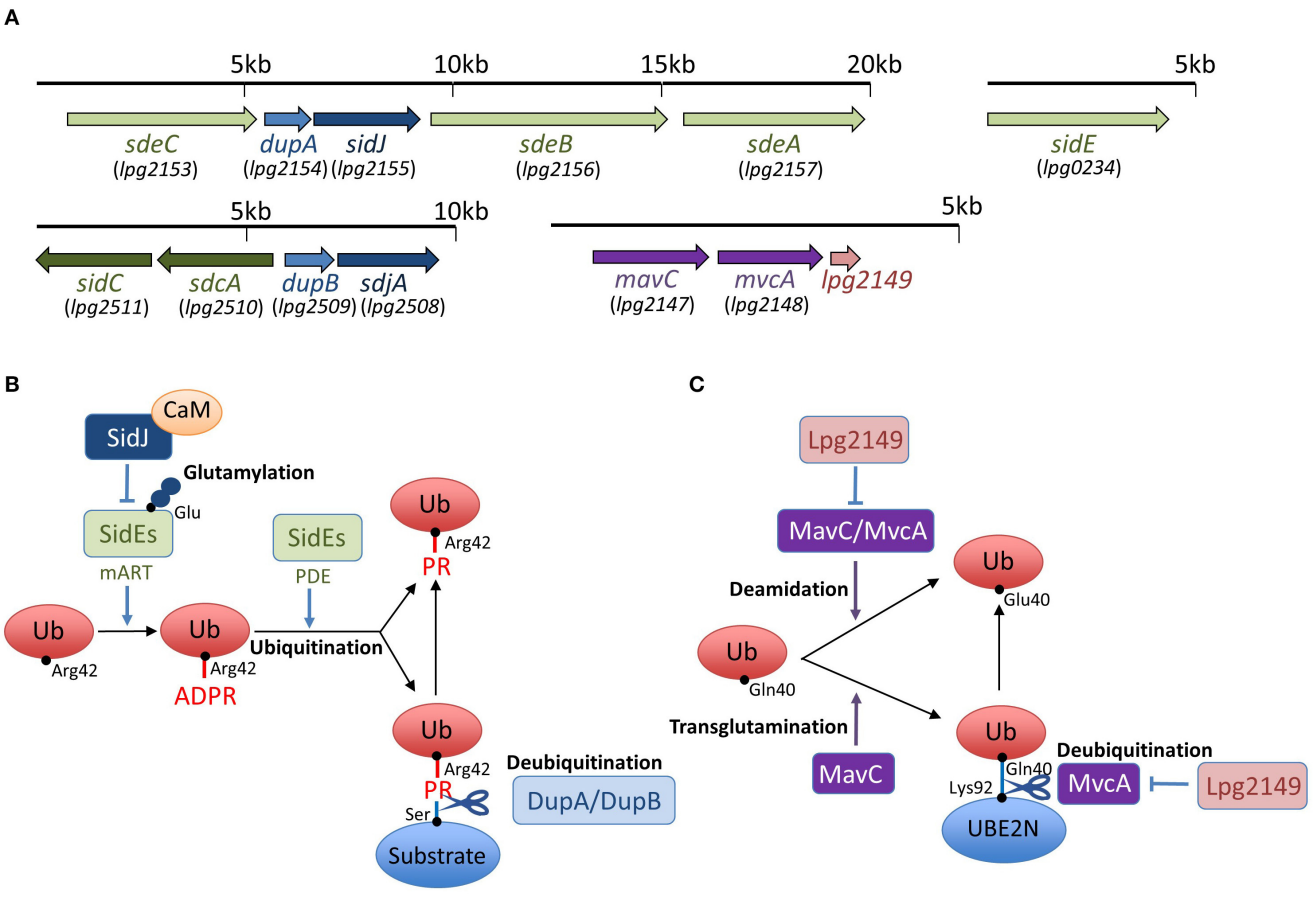
Schemes of effector interplay in the regulation of unconventional ubiquitination and gene organization of *sidEs, dupA/dupB, sidJ/sdjA*, and *mavC/mvcA* loci in *L*. *pneumophila*. **(A)** SidEs are encoded in two loci in the *L*. *pneumophila* genome. *sdeA, sdeB*, and *sdeC* genes are in a cluster, and the *sidE* gene is in a separate locus. DupA is encoded in the same locus as *sdeA, sdeB*, and *sdeC*, while DupB is encoded in a different locus. SidJ is encoded in a locus with *sdeA, sdeB, sdeC*, and *dupA*. *sdjA* is thought to be a paralogue of *sidJ*, representing in the same locus with *dupB*. *mavC* and *mvcA* are tandem-arrayed paralogous genes adjacent to *lpg2149*. **(B)** SidEs have an equivalent function in PR-ubiquitination mediated by mART and PDE activities. Products of the paralogous genes *dupA* and *dupB* can reverse PR-ubiquitination with their unique deubiquitination activity. Together with the cofactor CaM, SidJ executes an enzymatic effect to glutamylate the active site of the mART domain of SidEs, thereby inhibiting SidEs-mediated ubiquitination (see text for details). **(C)** MavC and MvcA display deamidation activity against ubiquitin. MavC, but not MvcA, possesses a transglutamination activity and forms a covalent bond between ubiquitin and the E2 enzyme UBE2N. The unconventional ubiquitination of UBE2N can be reversed by the unique DUB activity of MvcA. Lpg2149 can prevent the deamidase activity of both MavC and MvcA. It can also prevent the DUB activity of MvcA (see text for details).

**Figure 2:**
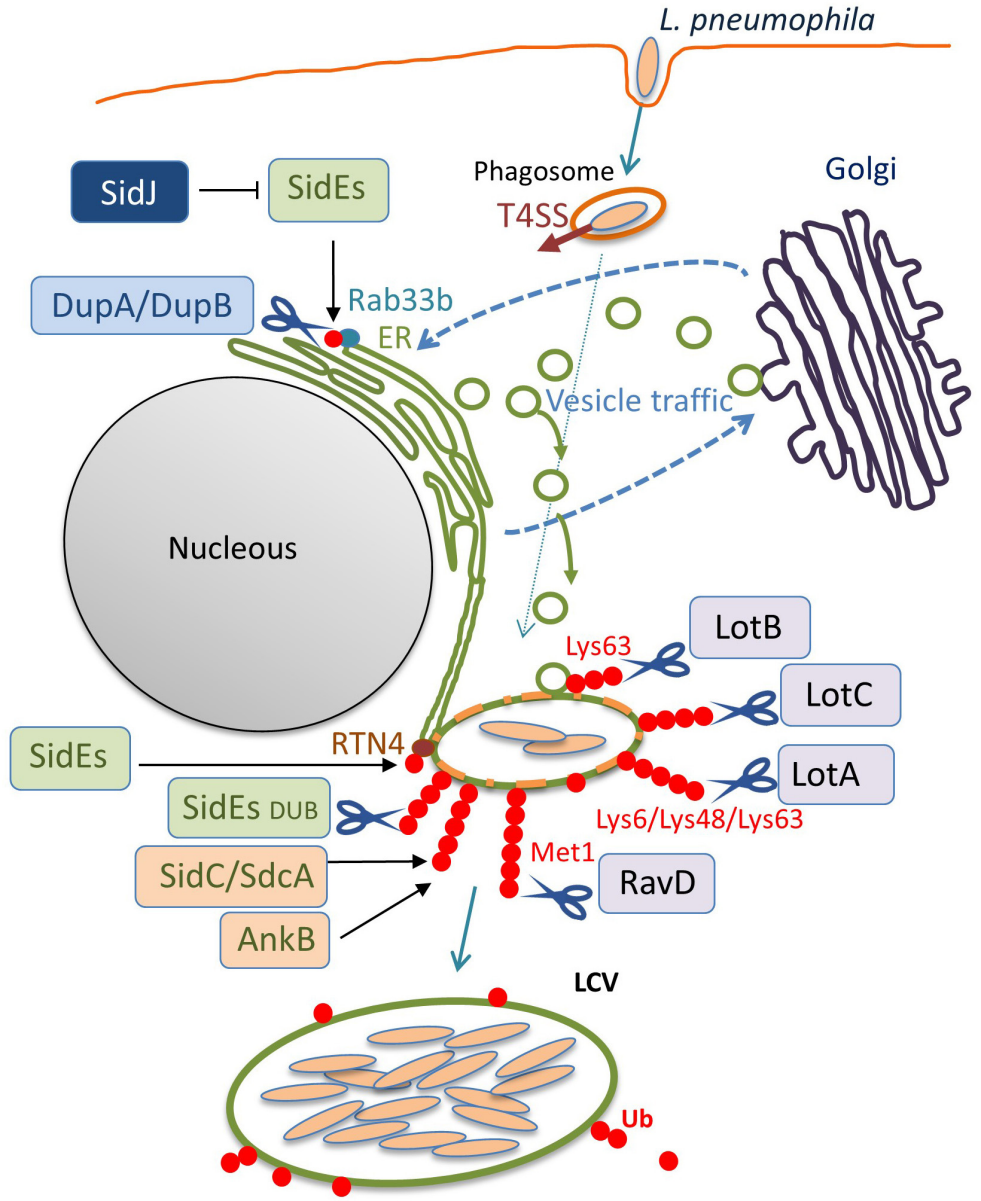
The ubiquitin system-modulating *L*. *pneumophila* effectors that are associated with LCV biogenesis. Once inside the host cell, *L*. *pneumophila* intercepts membrane traffic between the ER and the Golgi apparatus to acquire secretory vesicles to form the LCV. Among the *L*. *pneumophila* effector proteins delivered via the Dot/Icm T4SS, many enzymes associate with the LCV and catalyze ubiquitination, deubiquitination, or reactions that modulate the function of other effectors. In addition to the conventional ubiquitin ligases SidC, SdcA (Luo and Isberg, 2004; Ragaz et al., 2008; Hsu et al., 2014; Wasilko et al., 2018), and AnkB (Price et al., 2009, 2011), the unconventional ubiquitin ligases SidEs have been found to associate with the LCV. SidEs conjugate PR-Ub to many host proteins that function in the secretory pathway, such as Rab33b and RTN4, to modulate their functions. DupA and DupB remove the SidEs-conjugated PR-Ub from substrates with their DUB activity. SidJ negatively regulates the function of SidEs, using its enzymatic activity to modify the active site of SidEs. The amount of ubiquitin on LCVs can be reduced by the function of the bacterial DUBs LotA, LotB, LotC, and RavD. These can localize to the LCV and execute their function on the LCV, depending on their catalytic activities to cleave distinctive linkage-type ubiquitin chains. SidEs also act as canonical DUBs to remove ubiquitin from the LCV.

Interestingly, recent studies have discovered that *L*. *pneumophila* deploys a novel form of ubiquitination to establish LCV biogenesis (Figure 1). SidE, SdeA, SdeB, and SdeC are paralogues, known as the SidE family of proteins (SidEs). SdeA, SdeB, and SdeC are encoded in the same locus on the *L. pneumophila* chromosome, while SidE is encoded in a separate locus (Figure 1A). SidEs catalyze the ubiquitin ligation of host proteins with no requirement of E1 and E2 enzymes, which are essential for canonical ubiquitination (Qiu et al., 2016; Kotewicz et al., 2017). The reaction proceeds in two steps: first, a mono ADP-ribosyl transferase (mART) domain of SidE covalently attaches the ADP-ribose moiety of the cofactor nicotinamide adenine dinucleotide to the Arg42 residue of ubiquitin, resulting in the formation of an ADP-ribosylated ubiquitin (ADPR-Ub) intermediate. Then ADPR-Ub is catalyzed by a phosphodiesterase (PDE) domain of SidE to release AMP, resulting in the conversion to phospho-ribosylated ubiquitin (PR-Ub), which can attach to the serine residue of a substrate (Figure 1B). The substrates for PR-ubiquitination include reticulon 4 (RTN4) (Kotewicz et al., 2017) and Rab33b (Qiu et al., 2016). RTN4 is an ER-associated protein that regulates the dynamics of tubular ER (English et al., 2009). This protein is recruited to the LCV depending on the Dot/Icm T4SS (Haenssler et al., 2015; Kotewicz et al., 2017; Steiner et al., 2017). The enzymatic activity of SidEs is responsible for RTN4 recruitment to the LCV (Kalayil et al., 2018), and for ER remodeling as mediated by RTN4, which facilitates the initial step of LCV biogenesis (Figure 2) (Kotewicz et al., 2017). Rab33b is a small GTPase that is involved in retrograde transport from the Golgi apparatus to the ER (Starr et al., 2010). These mechanisms suggest that PR-ubiquitination mediated by SidEs is a key event in LCV formation.

Analyses of several *L*. *pneumophila* effector proteins have revealed a multi-tiered functional interaction among them. A class of proteins called metaeffectors has emerged; these are effectors that regulate other effectors (Kubori et al., 2010; Havey and Roy, 2015; Jeong et al., 2015; Urbanus et al., 2016; Shames et al., 2017; Valleau et al., 2018). In addition to the effector/metaeffector pairs, *L. pneumophila* possesses effector pairs with opposing activities against the same targets (Neunuebel et al., 2011; Tan and Luo, 2011; Tan et al., 2011). The existence of the effector/metaeffector and agonist/antagonist pairs provides another layer for the bacterial regulation of cellular processes. By utilizing metaeffectors, bacteria can finely regulate the activity of effectors in a temporal and spatial manner inside their host cells, striking a balance between perturbing cellular functions and maintaining a replicative niche (Kubori and Nagai, 2011; Hilbi et al., 2017). The first-identified metaeffector, LubX, is an E3 ubiquitin ligase that polyubiquitinates another *L*. *pneumophila* effector, SidH, and subjects it to proteasomal degradation (Kubori et al., 2008, 2010). Current knowledge indicates that, *L*. *pneumophila* possesses at least 10 proteins that have ubiquitin ligase activity (Qiu and Luo, 2017a), and a significant number of proteins have been inferred to function as deubiquitinases (DUBs), which have been rarely identified in other bacteria (Kubori et al., 2019). In this review, we highlight the fascinating role that *L*. *pneumophila* effectors play in reversing both conventional and unconventional ubiquitination in various ways to accomplish intracellular survival.

## SidE Family Proteins (SidE, SdeA, SdeB, and SdeC)

Sheedlo *et al*. utilized the suicide inhibitor of DUBs, the hemagglutinin-tagged ubiquitin vinyl methyl ester (HA-Ub-VME), which can form a covalent linkage with an active site of DUBs, to isolate *L*. *pneumophila* proteins that have potential DUB activity from bacterial lysates, which led to the first discovery of the *L*. *pneumophila* DUB, SdeA (Sheedlo et al., 2015). The crystal structures of the SdeA DUB module (SdeA_DUB_), both alone and in complex with Ub-VME, indicate that SdeA possesses a Cys–His–Asp catalytic triad. This triad is commonly found in the bacterial cysteine proteases belonging to the CE clan, which mainly consist of ubiquitin-like (Ubl) proteases (ULPs) and DUBs (Pruneda et al., 2016). Structural data indicate that SdeA_DUB_ most prominently engages Gln40 of ubiquitin, instead of the Ile44 patch that is widely used by eukaryotic DUBs for interaction, suggesting that some bacterial DUBs have evolved in different ways from eukaryotic DUBs, in terms of substrate preference. SdeA was shown to possess the ability to cleave Lys11/48/63-linked ubiquitin chains, with a distinct preference for Lys63-linkage. The architecture of the active-site cleft presents an open arrangement that has an advantage in accommodating varying structures of the three most abundant polyubiquitin chains. SdeA_DUB_ is also able to recognize and cleave the chains of neural precursor cell-expressed developmental downregulated 8 (NEDD8) but not of the Ubl protein small ubiquitin-related modifier 1 (SUMO1). Interestingly, structural similarity analyses of SdeA_DUB_ have identified the mammalian ULP family of cysteine protease Den1, which exhibits an exclusive specificity toward NEDD8. As an indicator of the functional significance of SdeA, polyubiquitin enrichment on *L*. *pneumophila* phagosomes is regulated by SdeA in infected mouse bone marrow-derived macrophages (Figure 2). The Cys–His–Asp catalytic triad is conserved among the DUB modules of SidE, SdeA, SdeB, and SdeC, and the DUB activity has been confirmed by *in vitro* reactions, not only for SdeA but also for SdeB and SdeC (Sheedlo et al., 2015).

In addition to the DUB activity of SdeA, it has been demonstrated that SdeA-mediated phosphoribosylation of ubiquitin impairs the conventional ubiquitination cascade (Bhogaraju et al., 2016). In an *in vitro* experimental system, the activities of the model E3 ubiquitin ligases, Parkin, tripartite motif-containing 56, and *Salmonella* effector protein SopA, were abrogated in the presence of SdeA, even without the DUB domain. Bhogaraju *et al*. reasoned that the apparent lack of activity among the E3 ligases was due to defective E1 or E2 reactions, because SdeA-modified ubiquitin cannot be effectively loaded onto E1 or E2 enzymes (Bhogaraju et al., 2016). They also demonstrated that cellular ubiquitin systems easily shut down when a pool of ubiquitin is utilized by SdeA. Therefore, it has been suggested that *L*. *pneumophila* can effectively control the overall ubiquitin systems in infected cells.

## DupA and DupB

Recently, two paralogues of *L*. *pneumophila* effectors, DupA and DupB, that possess the PDE domain, were identified as enzymes that can specifically reverse unconventional ubiquitination mediated by SidEs (Wan et al., 2019a; Shin et al., 2020b) (Figure 1B). The PDE domain is conserved in nine effectors of the Philadelphia strain of *L*. *pneumophila*; SidE, SdeA, SdeB, SdeC, DupA, DupB (SdeD), Lpg1496, Lpg2239, and Lpg2523 (Wan et al., 2019a). DupA and DupB are relatively small proteins, consisting of only the PDE domain. DupA is encoded by a gene located in the cluster *sdeA, sdeB*, and *sdeC* (Figure 1A). DupB was originally named SdeD, as a product of a *sidE* paralog, and it is encoded by a gene upstream of the paralogs *sdcA* and *sidC* genes, which encode the E3 ubiquitin ligases (Figure 1A).

The crystal structure of DupA has a resemblance to the PDE domain of SdeA (SdeA_PDE_) (Shin et al., 2020b). A comparison of the structure of SdeA_PDE_ to the crystal structures of DupB (SdeD) in complex with Ub and ADPR-Ub revealed that SdeA and DupB can interact with ubiquitin similarly using conserved residues (Akturk et al., 2018). Consistent with these facts, DupA and DupB have the ability to process ADPR-Ub to PR-Ub, as SidEs do. However, unlike SidEs, they do not transfer PR-Ub to the model substrate, Rab33b (Wan et al., 2019a; Shin et al., 2020b). Biochemical analyses revealed that these PDE domains exhibit hydrolase activity that cleaves the phosphoester bond between PR-Ub and the serine residue in the substrate (Figure 1B). In this sense, DupA and DupB are DUBs that remove PR-Ub from substrates specifically targeted by unconventional *L*. *pneumophila* ubiquitin ligases.

The enzymatic activities of DupA and DupB have been shown to suppress the Golgi fragmentation (Wan et al., 2019a) that has been reported to be caused by SdeA (Jeong et al., 2015). Furthermore, the proteomic identification of host proteins subjected to PR-ubiquitination was conducted utilizing a *dupA* and *dupB* double knock-out *L*. *pneumophila* strain (Wan et al., 2019a; Shin et al., 2020b). The identified proteins include Golgi reassembly-stacking protein 2, torsin-1A-interacting protein 2 (Wan et al., 2019a) and FAM134C, RTN1, RTN3, lunapark 1, and testis expressed 264 (Shin et al., 2020b), in addition to the previously reported RTN4 (Kotewicz et al., 2017). Validation of the PR-ubiquitination of the selected proteins has shown that multiple ER proteins are PR-ubiquitinated during *L*. *pneumophila* infection, which can contribute to LCV remodeling (Figure 2).

## MavC and MvcA

**Figure 3:**
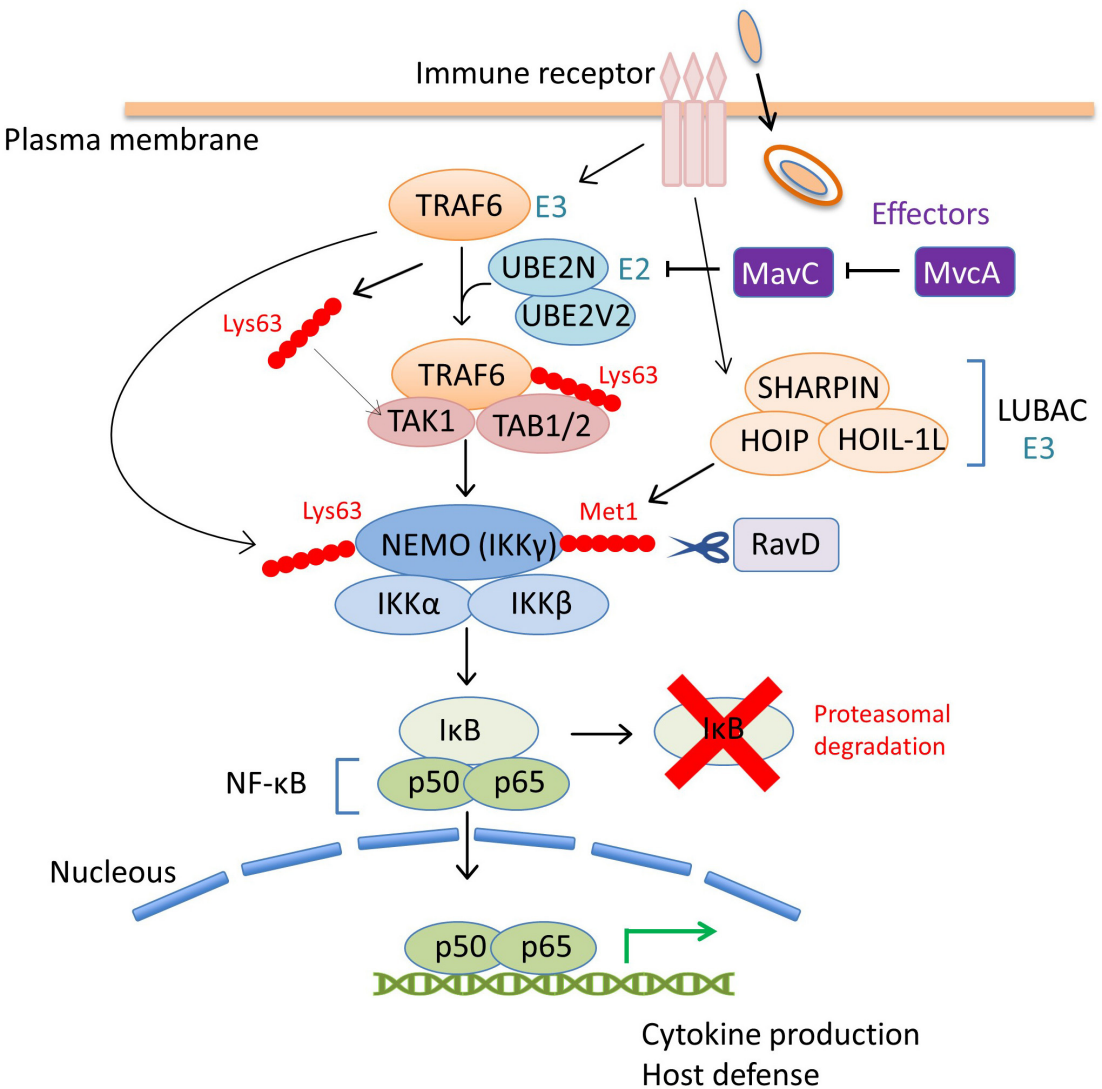
The coordinated regulation of the host NF-κB pathway by *L*. *pneumophila* effectors via ubiquitin modulation. Upon bacterial infection, the NF-κB family of transcription factors play a central role in controlling the host immune response, such as the production of proinflammatory cytokines, including tumor necrosis factor α (TNFα) and interleukin-1 (IL-1) and the induction of host defense signaling (Li and Verma, 2002). The inhibitor of NF-κB (IκB) suppresses the activity of NF-κB by binding and preventing nuclear translocation. The NF-κB pathway is mainly mediated by the engagement of host immune receptors that can recognize pathogen-associated molecular pattern molecules of extracellular or intracellular bacteria. One of the downstream pathways in which TNFα-receptor-associated factor 6 (TRAF6) is involved, is modulated by a *L*. *pneumophila* effector, MavC. TRAF6 is an E3 ubiquitin ligase that functions with the E2 enzyme hetero-dimer complex UBE2N/UBE2V2 (also known as Ubc13/Uev1A) to induce its auto-polyubiquitination and polyubiquitination of other neighboring proteins via Lys63-linked ubiquitin chains (Wang et al., 2012). The Lys63-linked ubiquitin chains of TRAF6 serve as docking sites for TGF-β-activated kinase 1 (TAK1)-binding protein 2 (TAB2). TAB1 and TAB2 promote the recruitment of the serine-threonine kinase TAK1 into the complex to activate the pathway. Activated TAK1 phosphorylates IκB kinase β (IKKβ) to promote the proteasomal degradation of IκB, thereby allowing NF-κB nuclear translocation. TRAF6-mediated Lys63-linked ubiquitin chains are associated with the NF-κB essential modifier (NEMO, also known as IKKγ) in the IKKα/IKKβ/NEMO complex and play a crucial role in IKK signaling. TRAF6 also produces free Lys63-linked polyubiquitin chains, which directly activate TAK1 kinase by binding to TAB2. In this scheme, the inhibitory activity of MavC against UBE2N dampens the NF-κB signaling pathway (including many other signaling branches not shown in this figure) by preventing overall Lys63-linked ubiquitination. MvcA, the metaeffector of MavC, can counteract the activity of MavC in the later stage of infection, presumably to restore cellular conditions that optimize bacterial replication. RavD, the other *L*. *pneumophila* effector, functions as a DUB, and contributes to negative regulation of NF-κB signaling. The host multi-subunit E3 ligase LUBAC, composed of HOIL-1L, HOIP, and SHARPIN, catalyzes the formation of Met1-linked linear ubiquitin chains. NEMO harbors a Met1 ubiquitin-specific binding domain that is important for NF-κB signaling (Komander et al., 2009; Rahighi et al., 2009). RavD, which is similar to OTULIN (Keusekotten et al., 2013), cleaves LUBAC-assembled linear ubiquitin chains, thereby inhibiting the host NF-κB response.

In addition to SidEs, MavC is another example of *L*. *pneumophila* effectors that catalyze non-canonical ubiquitination (Valleau et al., 2018; Gan et al., 2019a). MavC, a structural homolog of cycle inhibiting factor (Cif), a bacterial ubiquitin deamidase, reveals deamidase activity against ubiquitin but not against the Ubl protein NEDD8 (Valleau et al., 2018). This protein also catalyzes the covalent linkage of the Gln40 residue of ubiquitin to Lys92 or Lys94 residues of the host E2 enzyme UBE2N through its transglutaminase activity (Figure 1C). Thus MavC inhibits the enzymatic activity of UBE2N to form Lys63-linked ubiquitin chains that activate the nuclear factor κB (NF-κB) pathway (Gan et al., 2019a) (Figure 3). In this sense, MavC can be considered as a non-canonical ubiquitin ligase, and an enzyme that has inhibitory activity against UBE2N-mediated ubiquitination.

As can be seen in Figure 1A, *mvcA* is a *mavC* paralogue (50% amino-acid identity) (Figure 1A). Recently, Gan *et al*. identified the unique enzymatic activity of MvcA that hydrolyzes the Gln40-Lys92 isopeptide bond between ubiquitin and UBE2N that is formed by the transglutaminase activity of MavC (Gan et al., 2020) (Figure 1C). The prolonged inhibition of UBE2N activity by MavC-mediated transglutamination dampens the NF-κB pathway and restricts intracellular *L*. *pneumophila* replication (Gan et al., 2020). Gan *et al*. showed that MvcA functions as a specific DUB to restore UBE2N activity, thereby allowing NF-κB activation (Figure 3). Like MavC, MvcA possesses the catalytic cysteine residue required for ubiquitin deamidase activity (Valleau et al., 2018) (Figure 1C). The same residue Cys83 is required for the cleavage between ubiquitin and UBE2N. To gain structural insights into the unprecedented deubiquitination, the complex of MvcA_C83A_ and UBE2N-Ub (the wild-type MavC-catalyzed non-canonical ubiquitination product) was crystallized. Structural analyses revealed that the same catalytic center executes both deamidation and unique deubiquitination and that, as with MavC, the “insertion” domain of MvcA plays a vital role in the recognition of UBE2N-Ub (Gan et al., 2020). The insertion domain is not present in canonical ubiquitin deamidases such as Cif and a Cif homolog in *Burkholderia pseudomallei* (CHBP) (Yao et al., 2009; Valleau et al., 2018). It has been proposed that the insertion domain was acquired during evolution from the canonical ubiquitin deamidases to enable the ability to recognize the specific substrate (Gan et al., 2020).

Interestingly, the effector Lpg2149, which is encoded in a gene adjacent to *mavC* and *mvcA* (Figure 1A), is a metaeffector that negatively regulates the deamidase activity of both MavC and MvcA (Valleau et al., 2018) (Figure 1C). In combination with yeast two-hybrid analyses, the Lpg2149 structure, solved as a homodimer crystal, revealed a potential interface with MavC and MvcA, possibly masking catalytic residues (Valleau et al., 2018). It was further shown that Lpg2149 could inhibit the unique DUB activity of MvcA (Gan et al., 2020) (Figure 1C).

More recently, the MavC structure was extensively analyzed in three studies (Guan et al., 2020; Mu et al., 2020; Puvar et al., 2020). The importance of the insertion domain in MavC is highlighted in these studies in terms of the recognition of UBE2N. Guan *et al*. prepared the complex composed of the catalytic mutant of MavC (MavC_C74A_) and UBE2N-Ub. Solving the crystal structure of the MavC/UBE2N-Ub binary complex showed that the insertion and “tail” domains in MavC undergo rotational movement during the formation of the ternary complex, conferring the necessary flexibility to access UBE2N (Guan et al., 2020). They confirmed that the insertion domain is not essential for the ubiquitin deamidation activity of MavC but is essential for UBE2N ubiquitination. By comparison with the reported structures of MvcA (Valleau et al., 2018; Gan et al., 2019a), the structure of MavC/UBE2N-Ub revealed the distinctive features of MavC and MvcA in reactions, which explains why the highly similar proteins execute opposing functions. The residues in proximity to the active sites (Trp255 in MavC and Phe268 in MvcA) possess distinct properties that result in the differing affinity between the enzyme and the substrate. In MvcA, the lower binding affinity promotes the dissociation of the substrate, leading to deubiquitination. They reasoned, therefore, that transglutamination and deubiquitination mediated by MavC and MvcA, respectively, are forward and backward versions of the same catalytic reaction (Guan et al., 2020). Mu *et al*. also crystalized MavC_C74A_ with UBE2N-Ub (MavC/UBE2N-Ub binary complex) as well as the MavC/UBE2N/Ub ternary complex (Mu et al., 2020). Compared to the structure of the ternary complex, the binary complex structure revealed that the loop containing the UBE2N modification site, Lys92, undergoes a marked conformational change during ubiquitination, extending toward the deamidated Gln40 residue of ubiquitin. It has been suggested that this conformational change allows the reaction to be completed by MavC. They further analyzed the structure of MavC in complex with Lpg2149. This showed that Lpg2149 has an overlapping binding site with ubiquitin in MavC, suggesting that Lpg2149 can inhibit the ubiquitination of UBE2N by competing with ubiquitin to bind MavC.

Puvar et al. took a unique approach utilizing ubiquitin tethered to UBE2N via a disulfide bond (UBE2N-SS-Ub) that mimics the charged E2~Ub conjugate, the form of ubiquitin intrinsically present in the ubiquitin-reaction cascade (Puvar et al., 2020). Their biochemical and NMR-based interaction analyses showed that free ubiquitin binds only weakly to MavC, while UBE2N-SS-Ub has significantly higher affinity to MavC. This argues that MavC targets the UBE2N~Ub conjugate to catalyze an intramolecular transglutaminase reaction to form an isopeptide bond between UBE2N and ubiquitin. The crystal structure of the MavC/UBE2N-SS-Ub complex revealed that the conformational dynamics of the MavC insertion domain is crucial for the remodeling of the UBE2N active site. These results demonstrate that the mechanism of the MavC-mediated catalytic reaction underlies the protein dynamics that favor transamidation over deamidation. Considering that deamidated ubiquitin was undetected in cells infected with wild-type *L. pneumophila* (Valleau et al., 2018; Gan et al., 2019a), the ubiquitin deamidase activity associated with MavC and MvcA is not likely to be biologically significant. The reported analyses of MavC and MvcA give insights into the structural and functional divergence of the bacterial Cif family proteins.

## SidJ and SdjA

SidJ is one of a few *L*. *pneumophila* effectors that can result in substantial growth defects when deleted from the genome (Liu and Luo, 2007; Jeong et al., 2015). This protein is encoded in the same locus as *sde* genes (Figure 1A). The initial observation that SidJ can counteract the function of SidEs was reported by Jeong *et al*. The intracellular growth of *L*. *pneumophila* in *Acanthamoeba castellanii* was inhibited upon the over-expression of SdeA; however, this was only the case when SidJ was absent (Jeong et al., 2015). They found that the ectopic expression of *L*. *pneumophila* SidEs in both yeast and mammalian cells caused cellular growth defects, and toxicity was suppressed by co-expression of SidJ. From these results, they proposed that SidJ could function as a metaeffector that controls the activity of SidEs. Their study showed that SidJ could alter the cellular localization of SidE, resulting in the disappearance of SidE from the LCV.

Having observed that SidJ can suppress the yeast toxicity of SdeA, Qiu *et al*. conducted mutagenesis on SidJ to identify the residues responsible for this activity (Qiu et al., 2017). In relation to the critical residues Asp542 and Asp545, SidJ showed presumable enzymatic activity to interfere with the SdeA-mediated ubiquitination of Rab33b in eukaryotic cells. The DXXD motif has previously been shown to be required to counteract the cellular growth defect caused by SdeA expression and the displacement of SidE from the LCV (Jeong et al., 2015). It has also been demonstrated that SdeA_DUB_ does not remove ubiquitin from Rab33b. *In vitro* reactions using purified SidJ with di-ubiquitins (di-Ubs) or cellular ubiquitinated proteins suggested that SidJ has a canonical DUB activity. However, covalent binding between SidJ and Ub-VME was not detected, and potential catalytic cysteine residues were found not to be essential for the cleavage of ubiquitin chains (Qiu et al., 2017). These results suggested that SidJ does not possess DUB activity, or has a catalytic activity very different from that of classic DUBs. Further biochemical analyses by Qiu *et al*. revealed that SidJ, which was purified from *L*. *pneumophila*, has the ability to cleave the phosphodiester bond between PR-Ub and the serine residue of Rab33b, which is formed by SdeA (Qiu et al., 2017). Controversially, it was indicated that SidJ, purified from *Escherichia coli*, cannot hydrolyze PR-Ub from Rab33b (Bhogaraju et al., 2019; Black et al., 2019; Wan et al., 2019a). These results raised the possibility that SidJ isolated from *L. pneumophila* might associate with other proteins with DUB activity against PR-Ub.

Four independent studies opened the door to identifying the unexpected enzymatic activity of SidJ (Bhogaraju et al., 2019; Black et al., 2019; Gan et al., 2019b; Sulpizio et al., 2019). It has been shown that SidJ adopts a protein kinase fold. The putative kinase domain in SidJ is crucial for the inhibition of SdeA-catalyzed ubiquitination (Black et al., 2019). The key residues conserved in protein kinases, namely, a metal-binding residue Asp542 in the DXXD motif and an active-site ion-pair Lys367 residue, are both essential for the enzymatic activity of SidJ (Black et al., 2019; Sulpizio et al., 2019). However, an *in vitro* reaction using purified proteins and liquid chromatography-tandem mass spectrometry (LC-MS) has shown that the SidJ-dependent enzymatic modification of SdeA was not phosphorylation but glutamylation. LC-MS and mutagenetic analyses revealed that Glu860 is the major target residue in SdeA, which is the catalytic residue of mART activity (Bhogaraju et al., 2019; Black et al., 2019; Gan et al., 2019b; Sulpizio et al., 2019). Each of these studies has demonstrated that SidJ could catalyze the formation of isopeptide bonds between the amino group of glutamate and the γ-carboxyl group of the catalytic Glu860 residue in the mART domain of SdeA, thereby inhibiting SdeA-mediated PR-ubiquitination (Figure 1B).

The glutamylation activity of SidJ requires ATP/Mg^2+^ (Black et al., 2019). It was reported that an evolutionarily conserved pseudokinase is not “inactive,” but has the ability to transfer AMP from ATP to a protein substrate (AMPylation) (Sreelatha et al., 2018). Considering this remarkable finding, it is presumable that SidJ activates the glutamylation reaction, and is mediated by its AMPylation activity of the pseudokinase domain. Indeed, it was shown that SidJ undergoes self-AMPylation in the absence of glutamate or SdeA (Gan et al., 2019b). The crystal structure of SidJ captured the enzyme in complex with the AMP moiety (Gan et al., 2019b). The importance of the AMPylation reaction was demonstrated by structure-guided mutagenesis; mutations in residues involved in the binding of AMP abolished the activity of SidJ to inhibit the SdeA-mediated PR-ubiquitination (Gan et al., 2019b). The results of these studies suggest that the AMPylation activity of SidJ is primarily used for the activation of the reaction to glutamylate SdeA by adding an AMP moiety to the target Glu860 residue in SdeA, as proposed by Black et al. (2019), or by self-AMPylation of SidJ.

It was shown that Glu860 was polyglutamylated; Glu860 in SdeA was modified with a various number of glutamates, with two in the majority (Black et al., 2019). In addition, not only SdeA but also SdeB, SdeC and SidE were glutamylated by SidJ (Black et al., 2019). Interestingly, the glutamylation activity of SidJ requires an interaction with the host protein calmodulin (CaM) (Figure 1B). The structures of SidJ in complex with CaM determined by X-ray crystallography (Black et al., 2019; Gan et al., 2019b; Sulpizio et al., 2019) and cryo-electron microscopy (Bhogaraju et al., 2019) indicate that the interaction with CaM relies on the IQ motif located in the C-terminal region of SidJ. It is plausible that the requirement for the eukaryotic cofactor CaM is to ensure that inactivation of the SidEs would only occur in infected cells, but not in the bacteria where the proteins are untranslocated, as in the case of the adenylate cyclase toxin from *Bordetella pertussis* (Wolff et al., 1980). The CaM requirement also suggests that SidJ activity could be regulated by the dynamics of the intracellular Ca^2+^ concentration upon infection (Bhogaraju et al., 2019), but the significance of this remains to be fully elucidated.

In contrast with SidJ, its paralogue SdjA does not show any activity to rescue yeast toxicity or to affect the ubiquitination of Rab33b (Qiu et al., 2017), suggesting that SdjA has a different function from SidJ. The gene that encodes SdjA is located just downstream of *dupB*, which encodes the PDE protein that functions in the PR-Ub-specific DUB (Figure 1A). This fact suggests a possible linkage between SdjA and DupB in regulating PR-ubiquitination, although the role of SdjA has remained elusive.

## Lot-DUBs

Humans encode approximately 100 DUBs, 16 of which belong to the ovarian tumor (OTU) family, which displays great variation in structure and function (Du et al., 2019). The subfamily of OTU DUBs mediates important signaling cascades in eukaryotic cells, such as NF-κB signaling, interferon signaling, DNA damage repair, and immunity (Mevissen et al., 2013; Swatek and Komander, 2016). Unlike other DUBs, most OTU DUBs exhibit an intrinsic specificity against ubiquitin linkage types (Mevissen et al., 2013; Mevissen and Komander, 2017). For instance, otubain 1 preferentially cleaves Lys48-linked ubiquitin chains (Edelmann et al., 2009; Wang et al., 2009), while ovarian tumor deubiquitinase with linear linkage specificity (OTULIN, also known as FAM105B) exclusively cleaves Met1-linked linear ubiquitin chains (Keusekotten et al., 2013). Recent findings on OTU-family DUBs in *L*. *pneumophila*, Lot (*Legionella* OTU-like)-DUBs, have revealed the existence of a novel group of DUBs that are related to but functionally different from eukaryotic OTU proteins, as described in the following subsections.

### LotA

LotA (Lpg2248, also known as Lem21) was the first Lot-DUB identified among *L*. *pneumophila* Dot/Icm T4SS effectors (Kubori et al., 2018). The notable feature of LotA is the dual catalytic activity that it possesses; this protein has two distinctive catalytic cysteine residues, Cys13 and Cys303. Biochemical experiments demonstrated that Cys13 preferentially cleaves Lys6-linked ubiquitin chains, while Cys303 revealed a preference for polyubiquitin than di-Ubs. Infection analyses showed that the level of Lys48/Lys63-linked polyubiquitin chains decorated on LCVs was reduced predominantly by the enzymatic activity depending on C303 (Figure 2). Furthermore, LotA possesses the ability to bind to the early endosome-marker phosphatidylinositol 3-phosphate (PI(3)P) and the late endosome marker PI(3,5)P2 via the C-terminal region, and is located on the LCV throughout the early and late stages of infection. Mutagenetic analyses have found that the lipid-binding ability of LotA is crucial for both ubiquitin removal from- and localization to the LCV. Interestingly, a quintuple mutant strain lacking *lotA* and the four *sidEs* genes showed a significant growth defect relative to the quadruple mutant strain lacking only the four *sidEs* genes, while the *lotA* single-deletion mutant strain did not show a growth defect compared to the wild-type strain. Importantly, a LotA deficiency in PI(3)P-binding did not complement the growth defect of the quintuple mutant strain, but full-length LotA did. In addition, the catalytically inactive LotA C13S mutant, but not the C303S mutant, restored the growth defect. These data demonstrate that LotA plays an important role in *L*. *pneumophila* infection through lipid binding and deubiquitination of a protein decorated with polyubiquitin chains on the LCV. Further analyses exploring the target proteins for LotA should clarify its role in infection, as well as the biological significance of LotA in catalyzing the cleavage of Lys6-linked ubiquitin chains as a DUB. Structural analyses conducted with these points in mind would aid our understanding of the mechanisms by which LotA can differentially use two cysteine residues to execute its functions.

### LotB

LotB (Lpg1621, also known as Ceg23) is a recently characterized Lot-DUB that exhibits high specificity toward Lys63-linked ubiquitin chains (Ma et al., 2020; Schubert et al., 2020; Shin et al., 2020a). This protein was originally identified as a Dot/Icm T4SS effector regulated by the transcriptional activator PmrA (Zusman et al., 2007). However, neither its enzymatic activity nor physiological role had been elucidated. Recently, three independent research groups have analyzed its structure, and the catalytic motif in the N-terminal region is now seen as distantly similar to those associated with the members of the OTU protein subfamily (Ma et al., 2020; Schubert et al., 2020; Shin et al., 2020a). The crystal structure showed that the DUB domain of LotB has a papain-like OTU core fold with a typical catalytic triad, consisting of Asp–Cys–His (Ma et al., 2020). Intriguingly, structural analyses that compare it to eukaryotic OTU DUBs have shown that there is an inserted helical region that extends from the central catalytic Cys-loop (Ma et al., 2020; Schubert et al., 2020; Shin et al., 2020a). Interestingly, the recent report in a preprint suggests that the ubiquitin binding S1 site is located at the helical arm in this inserted region, and that LotB has an additional ubiquitin binding site (S1') that enables it to specifically cleave the Lys63-linked polyubiquitin chains (Shin et al., 2020a). It has also been shown that LotB has a potential dual catalytic activity against ubiquitin and SUMO1 (Schubert et al., 2020) and can bind itself to NEDD8 (Shin et al., 2020a).

The report additionally showed the co-localization of LotB with the resident-ER protein Calnexin, but not with the Golgi apparatus or with mitochondrial markers in the host cell, (Shin et al., 2020a). Importantly, LotB localizes to the LCV, and might regulate the proteins associated with Lys63-linked polyubiquitin chains on the LCV during infection (Ma et al., 2020) (Figure 2). Comprehensive MS-based interactome analyses have shown that LotB interacts with OTU deubiquitinase (OTUD) 4, which deubiquitinates K63-linked ubiquitin chains of myeloid differentiation primary response 88 (MYD88) and downregulates NF-κB-dependent inflammation (Shin et al., 2020a). Ma *et al*. explored whether LotB might influence the NF-κB signaling pathway and found that it does not associate with the pathway (Ma et al., 2020). Future identification of the host protein(s) targeted by the DUB activity of LotB could promote an understanding of the physiological role of LotB in *L*. *pneumophila* infection.

### LotC

Concomitantly with the analyses of LotB, another Lot-DUB LotC (Lpg2529, also known as Lem27) was also characterized in the studies recently reported in preprints (Liu et al., 2020; Shin et al., 2020a). The crystal structures of LotC (Shin et al., 2020a) and the LotC-ubiquitin-propargylamide (Ub-PA) complex (Liu et al., 2020) were solved, and revealed that LotC features the Asp–Cys–His catalytic triad, that is also present in LotB. The crystal structures revealed that, similar to LotB, LotC has an extended helical region between the Cys-loop and the variable loop, which is commonly shorter in eukaryotic OTU DUBs (Shin et al., 2020a), and that extensive hydrogen bonding can be used for the recognition of ubiquitin (Liu et al., 2020). However, LotC lacks the S1' site, which defines specificity in the Lys63-linked polyubiquitin chains in LotB (Shin et al., 2020a).

Liu et al. demonstrated that LotC (Lem27) localizes to the LCVs depending on the functional Dot/Icm T4SS and regulates the association of ubiquitin on the LCV (Liu et al., 2020) (Figure 2). Recently the host's small GTPase Rab10, which plays a vital role in *L. pneumophila* intracellular replication, was shown to be ubiquitinated and recruited to the LCV by the function of the E3 ligases, SidC and SdcA (Jeng et al., 2019). LotC (Lem27) was found to reverse the SidC-induced Rab10 ubiquitination and decrease its association with the LCV, showing the interplay between *L. pneumophila* ubiquitin ligases and a DUB (Liu et al., 2020). Intriguingly, Shin *et al*. determined that host proteins interact with catalytically inactive LotC in the presence or absence of the *L*. *pneumophila* ubiquitin ligases SidC and SdcA (Shin et al., 2020a). These results indicate that a significant number of ribosomal proteins interact with LotC in the presence of SdcA but not in the presence of SidC, suggesting distinct physiological roles for SidC and SdcA. Exhibiting 71% sequence similarity, SidC and SdcA had been thought to have an equivalent function, based on the observation that recruitment of Calnexin, which was impaired in the vacuoles containing SidC-deficient *L. longbeachae*, was restored either by *L. pneumophila* SidC or SdcA (Dolinsky et al., 2014).

### Common Features of Lot-DUBs

Among the three Lot-DUBs (LotA, LotB, and LotC), LotA is the only one without a solved three-dimensional structure. Notably, sequence analyses have shown that LotA also contains an extended region that LotB and LotC commonly possess (Shin et al., 2020a), which means that the three Lot-DUBs share a structural feature that distinguishes them from eukaryotic OTU DUBs. This raises the possibility that a novel class evolved from the eukaryotic OTU family. Like LotA (Kubori et al., 2018), LotC (Liu et al., 2020; Shin et al., 2020a) exhibits broad activity toward several polyubiquitin chain-types including Lys6-linkage. LotB (Ceg23) also possesses the Lys6-linkage preference with a weaker tendency (Schubert et al., 2020). In addition, another potential Lot-DUB, Ceg7 (Lpg0227), as well as OTU proteins encoded by other bacteria (*E. coli, Burkholdaria ambifaria, Rickettsia massiliae*, and *Wolbachia pipientis*) have a preference for the broad chain types, including Lys48-, Lys63-, K11- and Lys-6 linkages (Schubert et al., 2020). This implies that the evolution of the enzymes from eukaryotic OTU DUBs adapted to various types of ubiquitin chains and is not limited to *L. pneumophila* but widely occurs in pathogenic bacteria. An enterohemorrhagic *E. coli* (EHEC)-encoded HECT (homologous to E6AP carboxyl terminus)-like E3 ligase, NleL, is the only currently known bacterial enzyme that assembles Lys6-linked ubiquitin chains (Lin et al., 2011). NleL activity restricts the formation of actin pedestals under the adherent bacterium on the host cell surface (Lin et al., 2011) and enhances EHEC infection by ubiquitinating and inactivating JNKs (human c-Jun NH2-terminal kinases) (Sheng et al., 2017). Interestingly, NleL can assemble not only Lys6-linked but also Lys48-linked ubiquitin chains (Lin et al., 2011), and has the propensity to assemble branched ubiquitin chains (Hospenthal et al., 2013). Taken together, pathogenic bacteria are thought to have acquired the ability to finely regulate various types of ubiquitin conjugates. Atypical ubiquitin chains, including Lys6-linked polyubiquitin, may be detrimental to the intracellular survival of *L. pneumophila*; hence the evolution of Lot-DUBs, which have a multiplicity of enzymatic functions for adapting to specific circumstances, may have been induced by *L. pneumophila* infection.

## RavD

A recent exciting update regarding bacterial DUB is the discovery of RavD (Lpg0160), which specifically cleaves Met1-linked linear polyubiquitin chains (Wan et al., 2019b). Utilizing a unique assay, in which linear ubiquitin chains were reacted with lysates prepared from bacterial species of 19 different genera *in vitro*, Wan *et al*. found that only the lysate of the *L*. *pneumophila* strain Philadelphia 1 has the activity of cleaving linear ubiquitin chains (Wan et al., 2019b). Further *in vitro* DUB assays using purified proteins and the bacterial lysate of deletion mutant strains demonstrated that no other *L*. *pneumophila* effectors cleave linear ubiquitin chains. Sequence alignment analyses revealed that RavD orthologs are present in two different *Legionella* species, *L*. *clemsonensis* and *L*. *bozemanni*, in addition to nine *L*. *pneumophila* serogroups. The crystal structure of the N-terminal region of RavD from the *L*. *pneumophila* Corby strain, indicates that RavD adopts a papain-like fold with an unconventional Cys–His–Ser catalytic triad (Wan et al., 2019b). This triad is present in ubiquitin-specific proteases (USPs), including USP30, which shows Lys6-linkage specificity (Gersch et al., 2017). The conformation of linear di-Ubs in complex with RavD, as well as the RavD-interacting residues, are largely shared with those in complex with OTULIN, a linear-ubiquitin-specific host DUB. OTULIN negatively regulates the NF-κB signaling pathway, which is partly mediated by the host multi-subunit E3 ligase linear ubiquitin chain assembly complex (LUBAC), which is composed of heme-oxidized iron-responsive element-binding protein 2 ubiquitin ligase-1L (HOIL-1L), HOIL-1L-interacting protein (HOIP), and SHANK-associated RH-domain-interacting protein (SHARPIN) (Kirisako et al., 2006; Keusekotten et al., 2013) (Figure 3). However, the overall structure of RavD is distinct from that of OTULIN. Nevertheless, RavD has a function that closely resembles that of OTULIN, inhibiting host NF-κB signaling during infection (Figure 3). In addition, RavD localizes to the LCV and prevents the accumulation of linear ubiquitin chains on the LCV (Wan et al., 2019b) (Figure 2), which is implicated in the avoidance of xenophagy (Damgaard and Pruneda, 2019). Although the PI(3)P binding region at the C-terminus was predicted to be required for the localization of RavD to the LCV, this localization was independent from PI(3)P binding (Pike et al., 2019). This raises the question of whether RavD interacts with another membrane component to remain attached to the LCV.

## LupA

LupA (Lpg1148) is suggested to be a metaeffector that has the function of inactivating another *L*. *pneumophila* effector, LegC3. This protein silences the cytotoxicity caused by LegC3 in yeast (Urbanus et al., 2016). Urbanus *et al*. determined the crystal structure of LupA and identified the canonical Cys–His–Asp catalytic triad, suggesting that it is a plausible DUB (Urbanus et al., 2016). Using immunoprecipitation assays with the lysate of human cells co-expressing LupA and LegC3, ubiquitinated species of LegC3 were detected in the presence of catalytically inactive LupA variants, but not in the presence of wild-type LupA. This result shows that the plausible metaeffector activity of LupA, where it counteracts the function of LegC3, is mediated by its deubiquitination ability (Urbanus et al., 2016). However, unlike other reported effector-metaeffector pairs, LupA and LegC3 are not encoded in the same locus of the *L. pneumophila* chromosome. LegC3 is a glutamine (Q)-SNARE (soluble NSF attachment protein receptor)-like protein that interacts with host arginine (R)-SNARE vesicle-associated membrane protein 4, together with two other *L*. *pneumophila* Q-SNAREs, the effectors LegC7/YlfA and LegC2/YlfB, to hijack host vesicular trafficking for LCV remodeling during infection (De Felipe et al., 2008; Bennett et al., 2013; Yao et al., 2014; Shi et al., 2016). Investigating a mechanism by which the DUB activity of LupA against LegC3 contributes to the manipulation of host organelle trafficking could lead to interesting results.

## Discussion

*L*. *pneumophila* is a unique bacterial pathogen that possesses an extraordinary number of effector proteins with complicated functional networks in the infected host cells. Recent analyses of the *Legionella* effector proteins, focusing on the regulation of the ubiquitin systems, have found not only a mimicry of eukaryotic enzymes, but also many unprecedented chemical reactions. Here, we focused on *L*. *pneumophila* effector proteins, which are negative regulators of the ubiquitin system. Some of the identified *L*. *pneumophila* DUBs have the canonical catalytic triads that characterize the OTU superfamily proteases. However, structural analyses have found distinctive features of bacterial OTUs compared to the eukaryotic DUBs. One notable highlight in the field of *Legionella*-effector research is the discovery of non-canonical chemistries of ubiquitination, namely, SidEs mART/PDE-mediated PR-ubiquitination and MavC transglutaminase-mediated ubiquitination. Accordingly, reversing enzymes have been identified among the effectors, namely DupA/B, MvcA, and SidJ. These findings provide knowledge of unexpected enzymatic activities of bacterial effector proteins. In this series of analyses, structural and MS approaches have been adopted, which are powerful and indispensable tools for the identification of novel chemical reactions.

The studies examined in this review represent paradigms of the effector/metaeffector or opposing functional relationship. It is conceivable that, by utilizing these effector sets, *Legionella* can achieve fine-tuning of the cellular environment to maximize its own growth. The exact roles of the effectors discussed here remain to be fully explored. It can be expected that the functions of *Legionella* effectors characterized thus far are just the tip of the iceberg. More effectors with functional relevance for the host ubiquitination machinery are expected to be identified in the near future, including other genes that encode possible OTU domains and PDE domains. Further analyses will elucidate the comprehensive effector networks that regulate the ubiquitin system following the stages of *Legionella* infection.

## Author Contributions

TKu and TKi prepared the first draft of the manuscript. TKu prepared the figures and figure legends. HN and TKi discussed the contents and revised the manuscript. TKu revised and finalized the manuscript. All authors contributed to the article and approved the submitted version.

## Conflict of Interest

The authors declare that the research was conducted in the absence of any commercial or financial relationships that could be construed as a potential conflict of interest.
